# Effect of Modified Tapioca Starch on Mechanical, Thermal, and Morphological Properties of PBS Blends for Food Packaging

**DOI:** 10.3390/polym10111187

**Published:** 2018-10-25

**Authors:** Rafiqah S. Ayu, Abdan Khalina, Ahmad Saffian Harmaen, Khairul Zaman, Mohammad Jawaid, Ching Hao Lee

**Affiliations:** 1 Laboratory of Biocomposite Technology, Institute of Tropical Forestry and Forest Products (INTROP), Universiti Putra Malaysia, Serdang 43400 UPM, Selangor, Malaysia; ayu.rafiqah@yahoo.com (R.S.A.); jawaid@upm.edu.my (M.J.); 2 Laboratory of Biopolymer and Derivatives, Institute of Tropical Forestry and Forest Products (INTROP), Universiti Putra Malaysia, Serdang 43400 UPM, Selangor, Malaysia; 3 Institute of Tropical Forestry and Forest Products (INTROP), Universiti Putra Malaysia, Serdang 43400 UPM, Selangor, Malaysia; harmaen@upm.edu.my; 4 Polycomposite Sdn Bhd, Taman Kajang Sentral, Kajang 43000, Selangor, Malaysia; dr.khairulz@gmail.com

**Keywords:** polybutylene succinate, tapioca starch, mechanical properties, scanning electron microscopy, TGA

## Abstract

In this study, polybutylene succinate (PBS) was blended with five types of modified tapioca starch to investigate the effect of modified tapioca starch in PBS blends for food packaging by identifying its properties. Tensile and flexural properties of blends found deteriorated for insertion of starch. This is due to poor interface, higher void contents and hydrolytic degradation of hydrophilic starch. FTIR results show all starch/PBS blends are found with footprints of starch except OH stretching vibration which is absent in B40 blends. Besides, Broad O–H absorption in all specimens show that these are hydrogen bonded molecules and no free O–H bonding was found. SEM testing shows good interfacial bonding between PBS and starch except E40 blends. Therefore, poor results of E40 blends was expected. In TGA, a slightly weight loss found between 80 to 100 °C due to free water removal. Apart from this, insertion of all types of starch reduces thermal stability of blend. However, high crystallinity of starch/PBS blend observed better thermal stability but lower char yield. Starch A and B blends are suggested to be used as food wrap and food container materials while starch D blend is suitable for grocery plastic bags according to observed results.

## 1. Introduction

Food packaging is a frequent topic on global challenge as plastic packaging has largest demand especially developed countries that reliance on processed foods [1]. Conventional plastic packaging is a problematic material as it has good oxidation resistance, but creates landfill pollution [2]. Packaging materials that are made by synthetic polymers are widely used, even though they take a long time to decompose naturally, causing waste-product accumulation [3,4,5]. Therefore, it is necessary to substitute current packaging materials with biodegradable ones. The plastics used in food wrap and food containers may contact the food, therefore, edible plastic is highly recommended [6].

Starch is one of the most abundant low-cost natural resources and one of the important bioresources used in the food industry, such as paper, thickeners, and gelling agents [7]. Starch has good physical, mechanical, and barrier properties that have the potential to become active films. Nonetheless, limitations of starch are concerning for its hydrophilic nature and lack of antioxidant ability [8]. Therefore, modified starches are known as “environmentally friendly” materials and commonly used as stabilizers, viscosifying agent, coatings, and thickeners [9]. Modified starch is a chemically-altered food ingredient. The purpose of modifying the starch is to eliminate gluten content in starch and to improve its ability to keep the texture and structure of the food. The use of modified starch could reduce the processing time for compound formation. Proteins that exist in starch form networks and assist the enhanced plasticity and elasticity properties of the materials when preparing biopolymer-based packaging materials [10].

The use of tapioca starch as bioplastics in industrial food packaging is still limited due to its hydrophilic nature, brittleness, thermal instability, as well as high production cost and the large area of land needed to produce bioplastic [11]. Several technologies were found to improve the performance of starch bioplastics, such as plasticization, chemical modification, or blending with polyesters [12].

Attention to aliphatic polyester has greatly increased in recent years with regards to promising sustainable and biodegradable material prospects. Polybutylene succinate (PBS) is one of the biodegradable aliphatic polyesters that has been produced using petroleum-based monomers [13,14,15,16,17,18]. PBS polymer can be obtained from the polycondensation process of 1,4-butanediol and succinic acid [19]. PBS has virtuous physical and mechanical properties, good processability, and biodegradability for a wide range of applications [20]. PBS polymer is more stable under flowing nitrogen at temperatures below 220 °C with the rate of mass loss reaching a maximum at 390 °C, showing it has better thermal stability than PLA polymer (with a maximum at 365–385 °C) [21]. Additionally, the flexibility of PBS backbone and the presence of readily hydrolysable ester bonds, which are prone to catalytic degradation by microorganisms or enzymes, causing rapid degradation process for PBS polymer.

Starch/polyester blended plastics are meant to improve processing properties and better strength. Previous studies investigated two types of starch blended with PBS polymer [22]. It was found that higher starch content resulted in lower tensile strength, melt flow index, and higher moisture absorption for both starch/PBS blends. Moreover, conversion of starch into thermoplastic starch must be done by using glycerol before it blends with the PBS polymer [23,24]. This is totally unacceptable for commercialized use due to the longer production time and extra costs needed to be invested. Furthermore, glycerol content is another factor that influences performance. Higher glycerol content will shift the blends toward brittle region, yet it makes process easily, higher crystallinity content.

Modified tapioca starch can mix directly with PBS without undergoing the gelatanization process. It will fill the gap of knowledge by reducing the process period, as well as the production cost. In this study, the properties of modified tapioca starches/PBS blends will be studied and, most importantly, it is done by a simple fabrication method. This shall attract interest from researchers and industry, especially in food packaging. The PBS polymer simply blends with five different types of modified tapioca starch by using a rotating internal mixer and a hot press.

## 2. Experimental

### 2.1. Materials

PBS in the form of pallets were bought from PTT Public Company Limited in Bangkok, Thailand. The density of PBS is 1.26 g/cm^3^. Tapioca starch in the form of powder was obtained from PT Starch Solution in Karawang, Indonesia. It has five modified starch grades (A, B, C, D, E) with different properties. Table 1 shows its properties, as provided by supplier.

### 2.2. PBS and Starch Preparation

The PBS pallets are first dried in an oven at 80 °C to prevent excessive hydrolysis which can compromise the physical properties of the polymer. After that, PBS was melted in a counter-rotating internal mixer (Brabender, UPM, Serdang, Malaysia) at 115 °C with rotation of 60 RPM. Subsequently, modified tapioca starch (A/B/C/D/E) was loaded into the mixer and blended for another 10 min until a homogenous compound was achieved. The compound was crushed into small pieces by using crusher machine (UPM, Serdang, Malaysia) before being pressed. Next, the compound was molded by using a hot press at 115 °C for 4 min and left to cool for another 3 min. Then, specimens were cut into shapes according to specific characterization testing. Five specimens from every combinations will be tested for each conducted testing. The naming of specimens was done according to the type and the volume contents of starch in the PBS blends; for example, A40 represents 40 wt % of type A starch blended in PBS polymer. There are a total of 15 specimens constructed by five types of starch (A, B, C, D, and E) and three volume contents of starch (40, 50, and 60 wt %) in PBS polymer blends.

### 2.3. Characterization

#### 2.3.1. Mechanical Properties

The tensile test was carried out according to ASTM D638-14 test method at a strain rate 50 mm/min using an Instron universal tester (UPM, Serdang, Malaysia) under room temperature. For flexural testing was carried out under room temperature following the standard ASTM 790-17 standard. Optimum loadings of specimens were selected to conduct the following characterizations.

#### 2.3.2. Fourier Transform Infrared Spectroscopy

Fourier transform infrared spectroscopy (FTIR) was performed using Perkin Elmer 1600 infrared spectrometer (UPM, Serdang, Malaysia) to conduct in the wavenumber range of 500–4000 cm^−1^ with 4 cm^−1^ resolution. The positions of significant transmittance peaks at a wavenumber was tracked by using Nicolet software.

#### 2.3.3. Morphological Analysis

Morphology of the samples was observed using Hitachi S-3400N scanning electron microscope (SEM, UPM, Serdang, Malaysia) equipped with energy dispersive X-ray (EDX, UPM, Serdang, Malaysia) under an accelerating voltage of 15 kV. The samples were gold sputtered before observation to avoid the charging effect.

#### 2.3.4. Thermal Analysis

The thermal stability of the samples was characterized using a TA Instruments Q500 thermogravimetric analyzer, TGA, (UPM, Serdang, Malaysia). About 6 mg of sample was scanned from 30 to 900 °C at a heating rate of 20 °C·min^−1^ under nitrogen gas atmosphere.

## 3. Results and Discussion

### 3.1. Mechanical Properties

**Figure 1:**
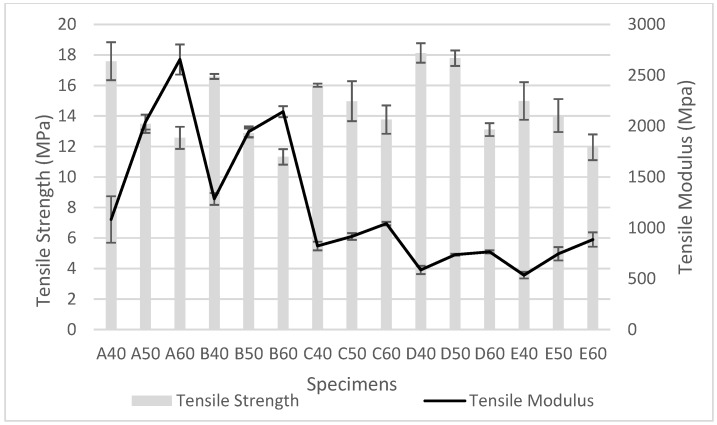
Tensile strength and tensile modulus of starch/PBS blends.

Mechanical properties of starch/PBS blends are presented in Table 2. Tensile strength and tensile modulus of the blends starch were compared in Figure 1. It is clearly shown that tensile stress for all types of starch were decreased as the volume content of starch increased. This could be explained by the main phase changing from PBS (comparatively flexible) to starch (comparatively rigid), reporting better rigidity, but lower strength and extensibility [22,25]. Additionally, low dispersion of starch in PBS resulted in the occurrence of stress concentration spots. Hence, deteriorated tensile strength is observed. In return, the tensile modulus is remarkably increased [26].

Apart from this, increasing starch content produces more voids, resulting in a weakening structure of the blends due to poorer interfacial bonding between PBS and starch. Starch E/PBS blends show the lowest average tensile values because of high chain branches of the amylopectin component richly found in starch E blends, which will detangled easily [16]. On the other hand, other starch blends showing better tensile strength were contributed from high crystallinity amylose content as stated in a previous work [22]. A high linear amylose structure in starch provided effective entanglement, which improved the tensile strength, and starch D shows the highest amylose contents with the highest tensile strength.

**Figure 2:**
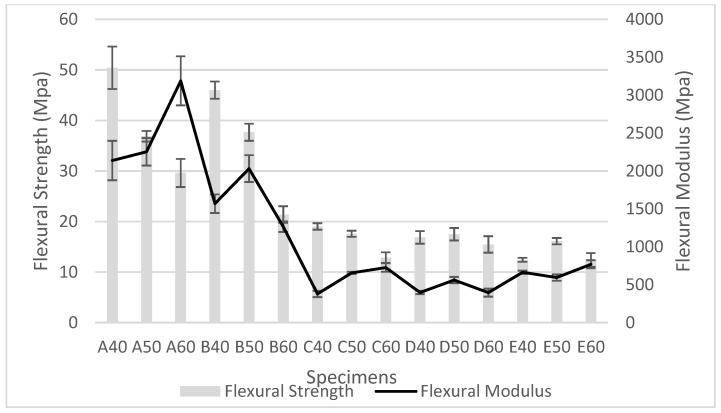
Flexural strength and flexural modulus of starch/PBS blends.

Flexural strength and flexural modulus of starch/PBS blends are given in Table 2 and its corresponding data were plotted in Figure 2. Overall, incrementing starch volume contents have reduced flexural strength of the blends. This could refer to the poor interface between the polymer and starch fillers, rendering the load transferring mechanism ineffective. As the loading of the hydrophilic starch increases, more hydroxyl groups induced hydrolytic degradation, ending in further deterioration of the flexural strength [15]. The lowest flexural strength found for the starch E blend was synchronized with other characterization testing.

Type D starch molecules tend to slip past each other under bending, leading to lower flexural modulus even though good tensile strength is observed [27]. On the other hand, 60 wt % of starch B and D in blends exceeded the optimum volume as more starch fillers slip away under the bending mechanism, indicating optimum filler insertion should be less than 50 wt % for starch B and D. In conclusion, the following characterization will be conducted on 40 wt % specimens due to budget constraints.

### 3.2. FTIR Analysis

**Figure 3:**
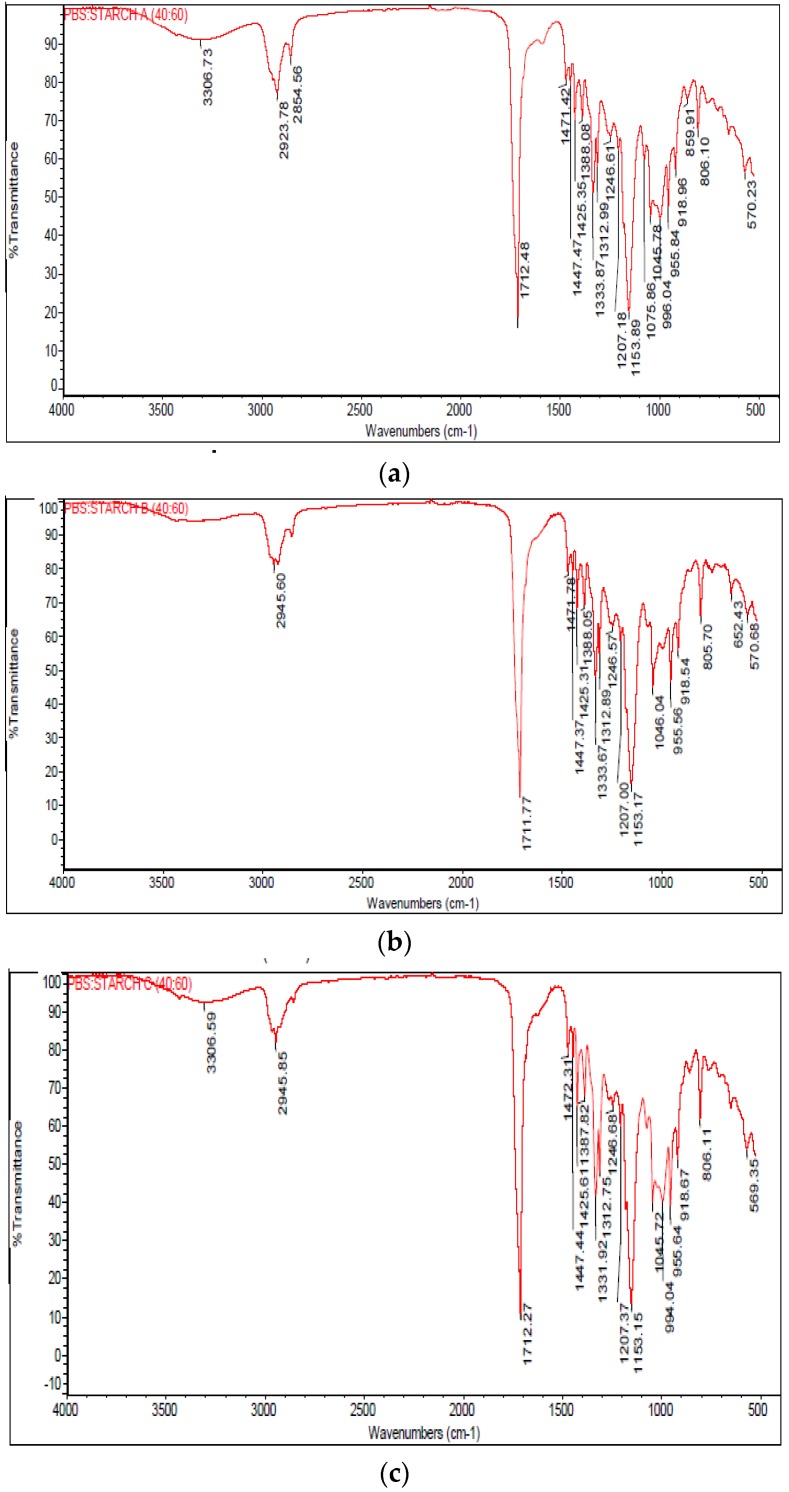

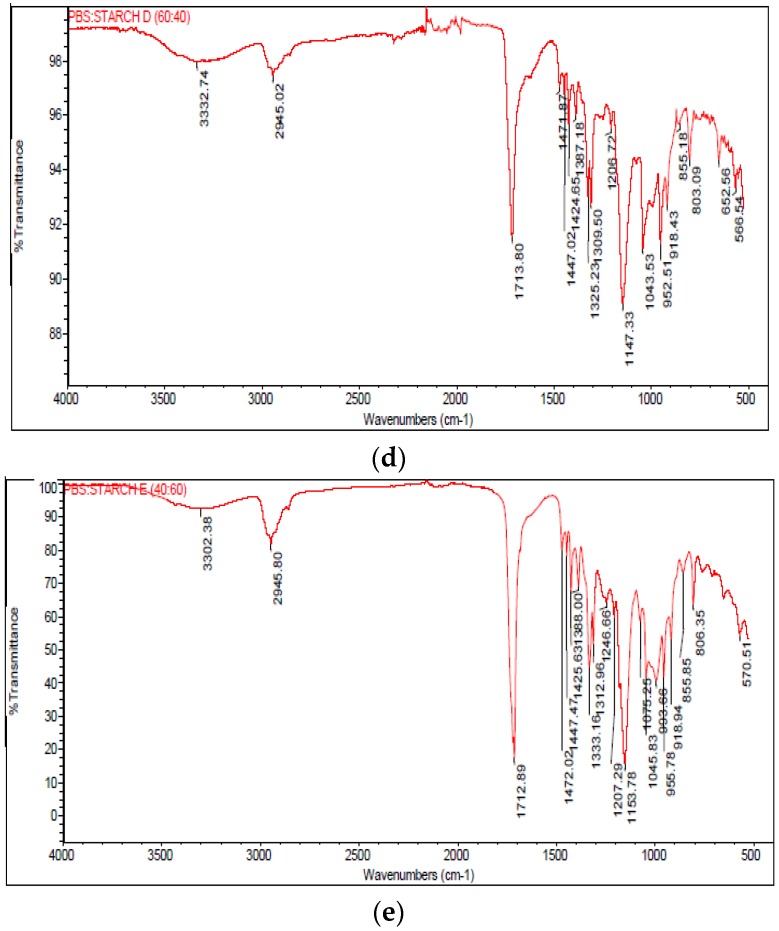
FTIR spectra of PBS blends with (**a**) Starch A; (**b**) Starch B; (**c**) Starch C; (**d**) Starch D; and (**e**) Starch E.

To study the interaction between starch and PBS polymer, FTIR spectra of the specimens were evaluated. Figure 3. shows the FTIR spectra of 40 wt % starch/BS blends. It is clearly visible at the starch fingerprints region of 800 to 1500 cm^−1^, that the spectral characteristic of all types of modified starch are similar. All starch/PBS blends have found footprints of starch which consist of alkene, esters, aromatics, ketone, and alcohol, with different oxygen-containing functional groups, e.g., OH (3400–3200 cm^−1^), C=O (1765–1715 cm^−1^), and C–O–(H) (1050 cm^−1^) excepted OH stretching vibration not found in sample B40. The O–H stretching mode is due to the hydrolytic effect of starch on the polymeric matrix, providing the formation of hydrogen bonded alcohol end groups [28]. Broad O–H absorption in all specimens shows that these are hydrogen-bonded molecules and no free O–H bonding was found. The presence of bonded hydrogen bonding also indicate stretching of water molecules strongly coupled to the structure of the starch and PBS, and the types of starch determined the strength of hydrogen bonds [29].

The band at 1156 cm^−1^ was associated with ordered structures of starch [30]. Amorphous regions of starch are mainly formed by internal long chains of amylose and amylopectin. In addition, amylose was found to be more concentrated at the periphery than in the core of the granule [31]. All spectra in the region below 800 cm^−1^ exhibiting complex vibrational modes due to the skeletal mode vibrations of the pyranose ring in the glucose unit.

PBS is a condensation biopolymer exhibiting hydroxyl and carboxyl terminated chains with the relevant end group content that is dependent on polymerization conditions [21]. Meanwhile, the region between 2800 and 3000 cm^−1^ represent the C–H stretch region. The peak in the 1710–1720 cm^−1^ region indicates the presence of C=O bonds in the presence of polybutylene succinate [32]. The region between 900 and 1149 cm^−1^ is associated with the presence of C–O bond stretching [33]. Additionally, the band located at 1153 cm^−1^ was associated with C–O bond flexion of the hydroxyl groups [34]. All spectra in the specific region were justified by the presence of PBS and starch materials in the specimen that was tested.

### 3.3. Morphology Analysis of Starch/PBS Blends

**Figure 4:**
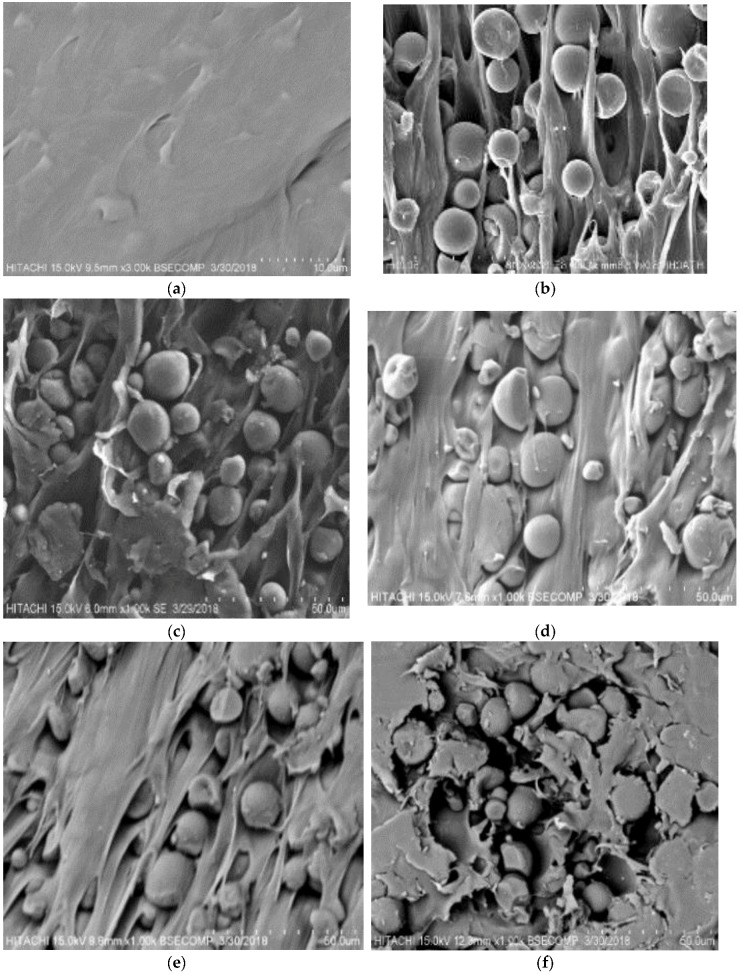
SEM analysis of (**a**) pure PBS; (**b**) A40; (**c**) B40; (**d**) C40; (**e**) D40; and (**f**) E40.

Mechanical properties are directly related to the morphology of composites. Cross-section cuts of the specimen variations were performed to explore the effects of different types of modified starches on the internal structure by SEM analysis (Figure 4). Figure 4a shows smooth and regular surfaces for the PBS polymer. Meanwhile, the surface for starch/PBS blends shows differently with various types of starch. The presence of modified tapioca starch resulted in a spherical shape embedded on the PBS surface [35]. Modified tapioca starch has a wider particle size distribution, toward the smaller diameters, as compared to its native starch. Starch A, B, C, and D blends show good interface with PBS compared with other types of starch. Other than that, they show homogenous morphology, resulting in strong interaction between starch and PBS polymer, further resulting in promising performance. This analysis was justified by the mechanical properties results. For Starch E, the image shows weak bonding between PBS and starch, therefore indicating easy debonding during fracture. Starch particles not well dispersed on the PBS surface cause weak bonding between the starch and PBS, which corresponds well with the results of tensile testing. A rough surface (Figure 4f) was discovered with some small holes and faults which are similar to previous studies of 40 wt % and above of PBS content [22,36,37].

### 3.4. Thermal Properties

**Figure 5:**
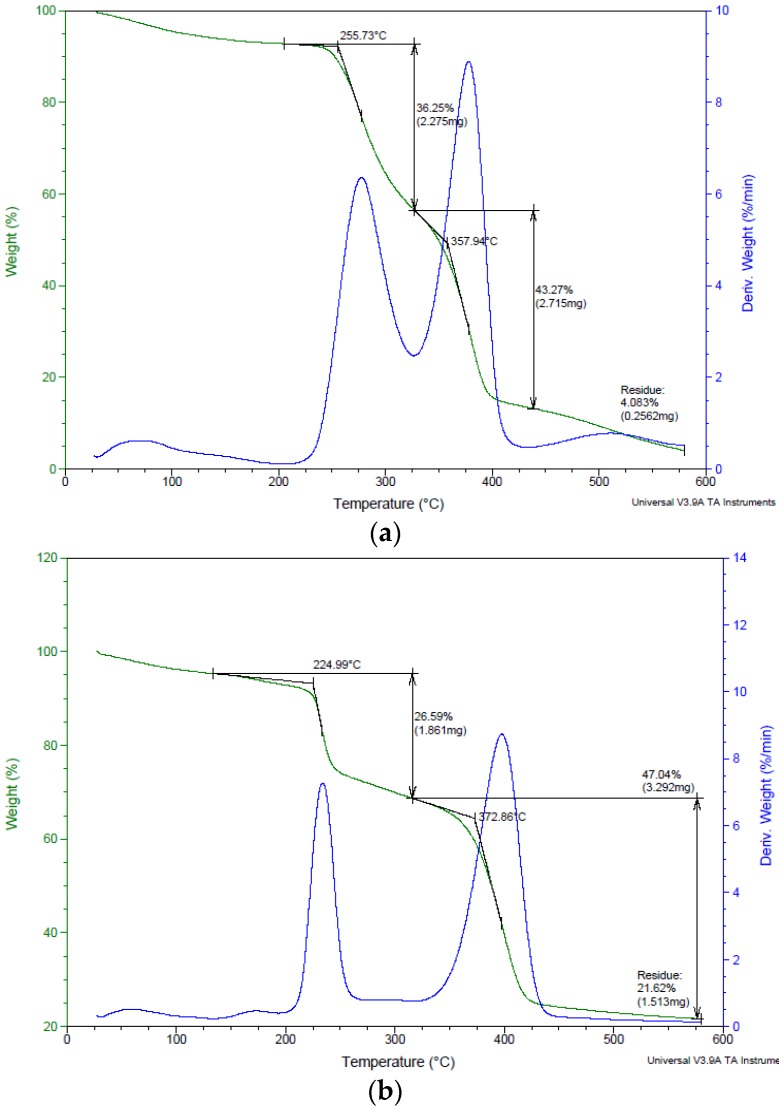

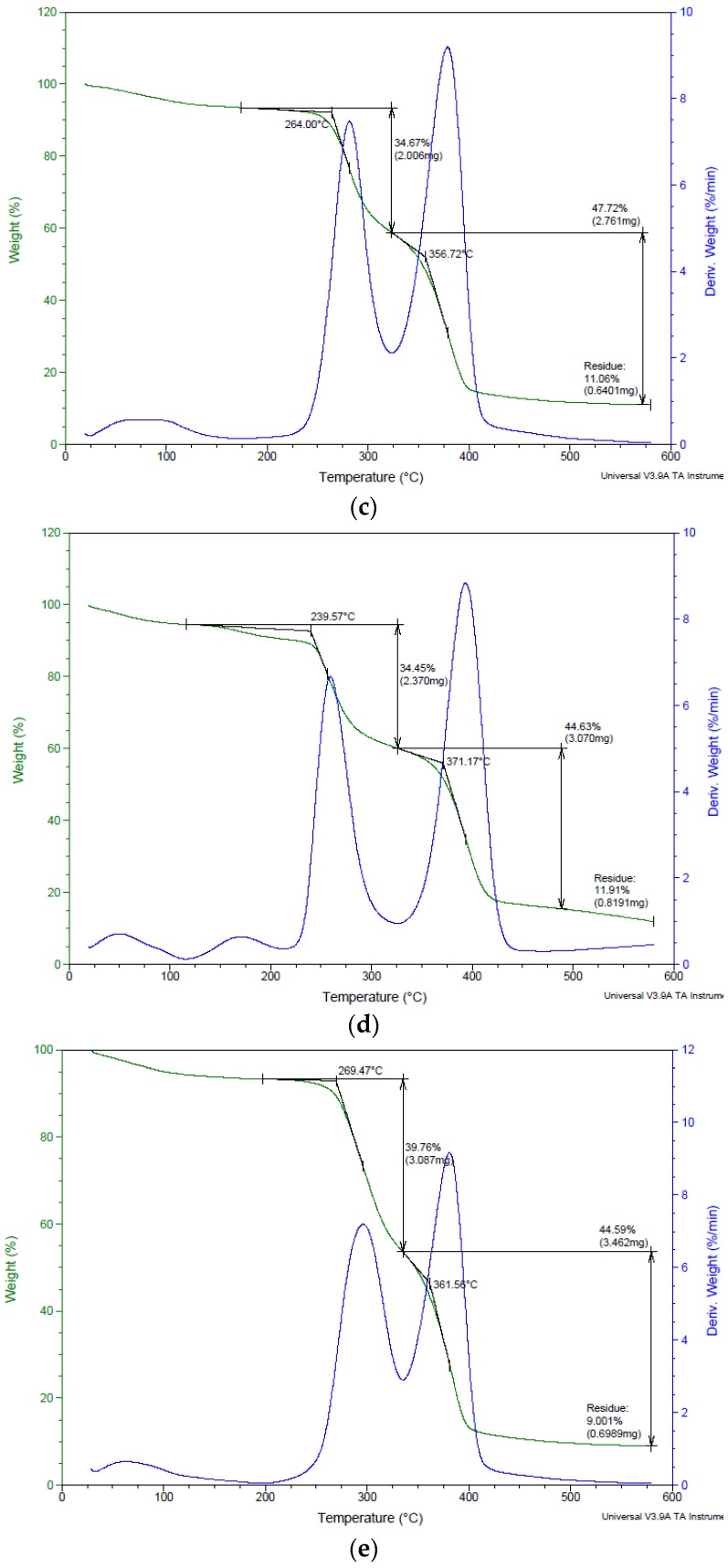
Analysis TGA for (**a**) A40; (**b**) B40; (**c**) C40; (**d**) D40; and (**e**) E40.

Thermal degradation of starch/PBS blends have been studied by TGA testing. Figure 5 shows the mass lost in every stage with peak temperature, as well as the residual mass at 600 °C. A slight weight loss was found between 80 and 100 °C for every specimen, reported due to water removal as starches have a high tendency to absorb moisture [38,39]. Thermal degradation of starch/PBS blends involved complex reactions, such as breakage of C–C, C–H, and C–O bonds, which showed in FTIR testing. The sharp transition occurring over a temperature range of 220–270 °C represents the decomposition of polysaccharide in starch/PBS blends. A transition which commenced at the onset temperature of about 356–372 °C can be attributed to the decomposition of the PBS. It is a highly thermally stable polyester (temperature for 5% weight loss = 338.14 °C, residual mass at 600 °C = 0.39%), therefore, the second mass loss reported belongs to the thermal degradation of PBS polymer. On the other hand, the first mass loss was the result of the degradation of starch [40].

Nonetheless, the insertion of all types of starch had observed lower onset temperature. This is due to the degradation of the blend beginning at the surface of the filler, which, in this study, referred to the starch. However, the main components in the starch are composed of high-crystallinity amylose and highly-branched amylopectin [41]. The higher the amylose content, the better the decomposition temperature. High crystallinity of starch (starch type A, B, C, and D, referred to good strength value) increase the thermal stability, but lowered the char yield as agreed by previous research [42,43]. Among all starch/PBS blend, the Starch D blend has the highest strength value, but opposed in the thermal decomposition temperature. This is consistent with the finding of Maubane (2017), high amylose content starch blends have the highest strength, but moderate thermal stability [16]. Additionally, better thermal stability was due to the low amount of remaining hydroxyl groups in the starch molecules after modification [44]. In this regard, the remaining weight at temperatures above 430 °C could be related to some residual solid and char attributed to the starch phase of the blend [45].

## 4. Conclusions

As a conclusion, insertion of modified tapioca starch into PBS polymer blends show better strength properties and thermal stability as compared to pure starch. However, the increment of starch content in blends observed a deteriorated property for all types of starch/PBS blends. Low dispersion of starch molecules, poor interfacial bonding, and high void content are responsible for the reduction in mechanical properties. Slipped molecules found under bending loads resulted in losing flexural capabilities. FTIR results show all starch/PBS blended are found with footprints of starch, except OH stretching vibration, which is absent in B40 blends. Additionally, broad O–H absorption in all specimens show that these are hydrogen-bonded molecules and no free O–H bonding was found. Moreover, SEM images show good interfacial bonding between PBS and starch, except sample E40. The poor interface between starch and PBS caused an increment in void content, weakening the structure of the blends and further reducing the mechanical properties. On the other hand, a slightly weight loss found between 80 and 100 °C was due to water removal from the hydrophilic nature of starch. Furthermore, insertion of all types of starches caused lower onset temperature, but higher residual char, first onset temperature, and second thermal degradation temperature are due to starch and PBS, respectively. The objective of this study is investigating properties of starch/PBS blends. All type of blends are possible to be used in the food packaging industry. Starch A/PBS and Starch B/PBS blends are applicable on food wrap and food container materials as they have very good elongation of break and outstanding bending capability. The Starch D/PBS blend has good tensile properties and is suitable to be used for plastic grocery bags.

## 5. Data Availability

The raw/processed data required to reproduce these findings cannot be shared at this time as the data also forms part of an ongoing study.

## Figures and Tables

**Table 1:** Properties of five starches.

Properties	Starch Type A	Starch Type B	Starch Type C	Starch Type D	Starch Type E
Moisture, %	11.1	8.1	7.2	8.6	11.2
Bulk Density, g/cm^3^	0.63	0.62	0.59	0.53	0.54
Beginning of gelatanization (*T*_g_), °C	51	45.2	44.9	60.3	69.4
Maximum Brabender viscosity, BU	1291	228	405	750	717
Viscosity, Cp	5.5	6.5	6.3	5.7	6.1

**Table 2:** Mechanical properties modified tapioca starch/PBS blends.

Specimen	Tensile Strength (MPa)	Tensile Modulus (MPa)	Flexural Strength (MPa)	Flexural Modulus (MPa)
A40	17.59 ± 1.25	1082.24 ± 228.21	50.41 ± 4.20	2137.70 ± 260.59
A50	13.49 ± 0.60	2040.13 ± 73.86	36.87 ± 1.06	2252.84 ± 181.69
A60	12.57 ± 0.72	2655.27 ± 148.21	29.61 ± 2.77	3188.48 ± 323.42
B40	16.59 ± 0.17	1284.82 ± 58.27	45.99 ± 1.70	1569.25 ± 122.82
B50	12.89 ± 0.330	1945.16 ± 52.01	37.67 ± 1.69	2031.28 ± 176.94
B60	11.32 ± 0.51	2142.13 ± 54.26	21.39 ± 1.64	1265.52 ± 69.70
C40	16.02 ± 0.11	821.46 ± 42.64	19.02 ± 0.64	378.690 ± 42.30
C50	14.97 ± 1.31	915.33 ± 32.98	17.58 ± 0.61	655.896 ± 14.63
C60	13.76 ± 0.93	1042.12 ± 17.42	12.87 ± 1.05	727.07 ± 55.79
D40	18.13 ± 0.64	586.48 ± 40.39	16.86 ± 1.25	397.87 ± 22.01
D50	17.79 ± 0.51	736.21 ± 10.12	17.49 ± 1.24	562.11 ± 40.65
D60	13.11 ± 0.42	764.39 ± 15.14	15.46 ± 1.63	395.57 ± 53.15
E40	14.98 ± 1.23	534.77 ± 32.47	12.43 ± 0.40	663.304 ± 21.69
E50	14.03 ± 1.08	745.18 ± 65.94	16.12 ± 0.62	594.282 ± 42.28
E60	11.95 ± 0.84	885.01 ± 70.50	12.40 ± 1.35	770.02 ± 53.62
